# Prevalence and Predictors of Subclinical Micronutrient Deficiency in German Older Adults: Results from the Population-Based KORA-Age Study

**DOI:** 10.3390/nu9121276

**Published:** 2017-11-23

**Authors:** Romy Conzade, Wolfgang Koenig, Margit Heier, Andrea Schneider, Eva Grill, Annette Peters, Barbara Thorand

**Affiliations:** 1 Helmholtz Zentrum München, German Research Center for Environmental Health (GmbH), Institute of Epidemiology II, Ingolstädter Landtraße 1, 85764 Neuherberg, Germany; romy.conzade@helmholtz-muenchen.de (R.C.); heier@helmholtz-muenchen.de (M.H.); shneider@helmholtz-muenchen.de (A.S.); peters@helmholtz-muenchen.de (A.P.); 2 German Heart Centre Munich, Technical University of Munich, Lazarettstraße 36, 80636 Munich, Germany; koenig@dhm.mhn.de or wolfgang.koenig@uniklinik-ulm.de; 3 DZHK (German Centre for Cardiovascular Research), Partner Site Munich Heart Alliance, 80802 Munich, Germany; 4 Ulm Medical Center, Department of Internal Medicine II—Cardiology, University Hospital Ulm, Albert-Einstein-Allee 23, 89081 Ulm, Germany; 5 Central Hospital of Augsburg, MONICA/KORA Myocardial Infarction Registry, Stenglinstraße 2, 86156 Augsburg, Germany; 6 Institute for Medical Information Processing, Biometrics and Epidemiology, Ludwig-Maximilians-University Munich, Marchioninistraße 15, 81377 Munich, Germany; eva.grill@med.uni-muenchen.de

**Keywords:** subclinical micronutrient deficiency, vitamin D, folate, vitamin B_12_, iron, predictors, older adults, population-based study, Germany

## Abstract

Subclinical micronutrient deficiency in older adults is associated with chronic age-related diseases and adverse functional outcomes. In Germany, the older population is at risk of insufficient micronutrient intake, but representative studies on micronutrient status in old and very old adults are scarce. This study’s objectives were to estimate the prevalence of subclinical vitamin D, folate, vitamin B_12_ and iron deficiencies among older adults, aged 65 to 93, from the KORA-Age study in Augsburg, Germany (*n* = 1079), and to examine associated predictors, using multiple logistic regression. Serum concentrations of 25-hydroxyvitamin D (25OHD), folate, vitamin B_12_, and iron were analyzed. The prevalence of subclinical vitamin D and vitamin B_12_ deficiencies were high, with 52.0% and 27.3% of individuals having low 25OHD (<50 nmol/L) and low vitamin B_12_ concentrations (<221 pmol/L), respectively. Furthermore, 11.0% had low iron (men <11.6 µmol/L, women <9.0 µmol/L) and 8.7% had low folate levels (<13.6 nmol/L). Common predictors associated with subclinical micronutrient deficiency included very old age, physical inactivity, frailty and no/irregular use of supplements. Subclinical micronutrient deficiency is a public health concern among KORA-Age participants, especially for vitamins D and B_12_. The predictors identified provide further rationale for screening high-risk subgroups and developing targeted public health interventions to tackle prevailing micronutrient inadequacies among older adults.

## 1. Introduction

Deficiencies in essential vitamins, minerals and trace elements (micronutrients) affect an estimated 2 billion people, in both developing and developed countries. This ‘hidden hunger’ has negative health impacts among vulnerable groups in the population, especially older adults [1].

Epidemiological evidence suggests that subclinical micronutrient deficiencies in older adults are associated with chronic age-related diseases and adverse functional outcomes. Older adults with low 25-hydroxyvitamin D (25OHD) levels have a significantly higher risk for type 2 diabetes mellitus, cardiovascular disease (CVD) and osteoporosis-related fractures [2]. Age-associated declines in muscle mass and strength (sarcopenia), which in turn affect balance, gait, and overall independence [3], have been linked to low 25OHD levels as well [4]. Vitamin B_12_ and folate are necessary for one-carbon metabolism and DNA synthesis, and have been investigated in relation to degenerative diseases, including CVD, cognitive dysfunction and osteoporosis [5]. Iron deficiency is the most prevalent nutritional deficiency worldwide and its main consequence is anemia [6], which has been associated with an increased risk of developing dementia in old age [7].

Ageing is associated with physiological changes that can impact the nutritional and more specifically the micronutrient status of older adults. Importantly, energy requirements decrease due to a loss of lean body mass and a reduction in physical activity, resulting in decreasing energy intakes. At a low energy intake, older adults may be at increased risk for specific micronutrient deficiencies, unless their diets contain micronutrient-rich foods. Moreover, older adults’ abilities to absorb and utilize specific micronutrients become less efficient (e.g., reduced gastric acid secretion preventing the release of vitamin B_12_ from foods [5]; decline in subcutaneous vitamin D synthesis capacity and lowered renal conversion to its active form [8]). Last, but not least, the likelihood of multiple chronic diseases, such as cardiovascular disease, type 2 diabetes mellitus and cancer, use of multiple medications and variable diet quality, associated with appetite loss, chewing/swallowing difficulties and socioeconomic barriers, may affect and even increase requirements for specific micronutrients [9,10]. All these factors make it difficult to ensure an optimal micronutrient supply for the older population.

Due to the role of micronutrient status in chronic disease and health prevention, it is important to quantify the magnitude of subclinical micronutrient deficiency and to identify subgroups at risk in the older population. Results from the second German National Nutrition Survey (NVS II) uncovered a high prevalence of insufficient dietary intake of vitamin D, folic acid and calcium in older adults aged 65 years and over. Vitamins B_1_, B_2_, B_12_ and iron were additional critical micronutrients in older women [11]. More recently, the national ‘German Health Interview and Examination Survey for Adults’ (DEGS1) revealed that 69.9% of women and 62.6% of men, aged 65 to 79, had low 25OHD <50 nmol/L [12], signaling a potentially serious public health issue. Nationally representative studies on the statuses of other micronutrients, in old and very old adults, in Germany, are scarce.

To support health care in developing purposive prevention measures that handle micronutrient inadequacies among older adults, factors associated with subclinical micronutrient deficiencies in older adults have to be identified. Besides insufficient dietary intake and impaired absorption, low micronutrient levels in older adults have been studied in relation to increasing age, female sex, poor eating habits, low physical activity, smoking, obesity, low kidney function, presence of chronic diseases, intake of drugs, non-use of supplements and, specifically for vitamin D, low sunlight exposure and winter season [13,14,15,16,17,18]. Despite recent concerns about the possible high prevalence of subclinical micronutrient deficiencies in the German older population, and apart from vitamin D [12,18], there is a lack of data on both micronutrient status and its predictors in older adults.

In light of the growing ageing population, as well as the high societal relevance of successful healthy ageing, this cross-sectional study aimed to provide insight into the magnitude of subclinical micronutrient deficiency in German older adults, aged 65 and over, from the population-based KORA (Cooperative Health Research in the Region of Augsburg)-Age study. Based on the micronutrients identified in the NVS II as possible public health concerns in the German older population, we selected four critical micronutrients with available nutritional biomarkers in the KORA-Age dataset. Specific objectives were (i) to determine the prevalence of subclinical vitamin D, folic acid, vitamin B_12_ and iron deficiencies in this older population, using serum biochemical biomarkers; and (ii) to identify predictors of subclinical micronutrient deficiency using multiple logistic regression. Possible predictors examined included socio-demographic, lifestyle, and health factors, as well as the use of supplements containing specific micronutrients.

## 2. Materials and Methods

### 2.1. Study Design and Participants

Data for the present analysis was obtained from the population-based KORA-Age study, conducted in 2008/2009, which was a follow-up of all participants, aged 65 and over on 31 December 2008 (*n* = 9197), who took part in at least one of four cross-sectional MONICA (Monitoring of Trends and Determinants in Cardiovascular Disease)/KORA health surveys S1–S4 conducted between 1984 and 2001, among the inhabitants of Augsburg and surrounding counties. Details about the general study design and participants have been described previously [19]. Briefly, a self-administered health questionnaire was mailed to all eligible participants of the KORA-Age cohort, i.e., those who were still alive and reachable in 2008/2009 (*n* = 5991). The response rate was 76.2% (*n* = 4565). Moreover, 68.9% of eligible participants took part in a standardized telephone interview (*n* = 4127). The present analysis refers to a sex and age-stratified random sample of *n* = 2005 eligible individuals of whom *n* = 1079 (537 men, 542 women) participated in an extensive physical examination in 2009 (response rate 53.8%). All examinations were performed by trained interviewers. A flowchart of the KORA-Age 2008/2009 recruitment and retention profile is shown in Supplementary Figure S1.

### 2.2. Ethical Considerations

Prior to their inclusion in the study, written informed consent was obtained from all participants or from the patient’s caregiver when the participant was unable to make an informed decision. The Ethics Committee of the Bavarian Medical Association (Bayerische Landesärtzekammer) approved the study protocol (date of approval: 11 November 2008, reference number: 08064).

### 2.3. Blood Sample Processing

Non-fasting blood samples were collected between February and November 2009 at the KORA study center and drawn into serum gel S-Monovette tubes (Sarstedt, Nümbrecht, Germany). Blood was gently inverted twice and rested for 30 min at room temperature until complete coagulation. After centrifugation at 15 °C for 10 min, the serum obtained was aliquoted into Nunc cryotubes (Thermo Fisher Scientific, Waltham, MA, USA). For the analysis of iron status, serum probes were kept at 4 °C for a maximum of 6 h and directly analyzed at the central laboratory of Augsburg Hospital. For vitamin D, folic acid and vitamin B_12_ status, serum probes were frozen at −80 °C at the KORA study center, transported on ice and stored at a minimum of −80 °C until analysis, in partner laboratories, between August and September 2011. Months of blood collection were categorized according to calendar seasons: spring (February–May), summer (June–August) and autumn (September–November).

### 2.4. Biochemical Analyses of Nutritional Biomarkers

Serum concentrations of 25-hydroxyvitamin D (25OHD), folate and cobalamin (vitamin B_12_) were measured by an electrochemiluminescence immunoassay (ECLIA, Elecsys 2010, Roche Diagnostics GmbH, Mannheim, Germany). The intra- and inter-assay coefficients of variations were 4.9% and <10% for 25OHD, 7.0% and <10% for folate and 5.3% and <10% for vitamin B_12_. Iron levels were measured by photometric measurements, using the chromophore Ferene^®^ (Dimension^®^ Iron Flex^®^ reagent cartridge, Dade Behring, Inc., Newark, DE, USA). The inter-assay coefficient of variation was <10%, and the maximal permissible imprecision and inaccuracy were 4% and 6%, respectively.

### 2.5. Cut-Off Points to Classify Subclinical Micronutrient Deficiency

Exact cut-off points for classifying subclinical micronutrient deficiencies remain debated. Recently, a serum 25OHD level of ≥50 nmol/L was recommended as an indicator of optimal vitamin D status by the critical review of the German Nutrition Society (DGE) for DACH countries (Germany, Austria and Switzerland) [20] as well as the last Nordic Nutrition Recommendations (NNR 2012) [21]. Accordingly, subclinical vitamin D deficiency was defined as a serum 25OHD level of <50 nmol/L. Subclinical folate and vitamin B_12_ deficiencies were defined as serum folate <13.6 nmol/L [22] and serum vitamin B_12_ <221 pmol/L [23], respectively. For serum iron, cut-offs were <11.6 µmol/L for men and <9.0 µmol/L for women [24].

### 2.6. Assesment of Predictors

The selection of potential predictors of subclinical micronutrient deficiencies was informed by the literature and their availability in the KORA-Age dataset. Variables were grouped into three categories (socio-demographic, lifestyle, health factors) plus the season of blood collection for vitamin D. Assessment methods and categorization of variables are described in Supplementary Table S1.

Briefly, the variables—sex, age groups and family status—were collected using the short form of the Demographics Standards of the German Society of Epidemiology [25]. Educational attainment was estimated by recording years of school completed. With the Geriatric Nutritional Risk Index (GNRI), the risk for malnutrition was assessed by measuring albumin, weight and height [26]. The Nutrition Score, indicating the risk of general malnutrition, was calculated using the German short form of the SCREEN II (Seniors in the Community: Risk Evaluation for Eating and Nutrition, version II) questionnaire [27]. The physical activity assessment included the frequency and duration of activity in summer and in winter [28]. Alcohol intake was assessed as daily average intake, based on the last weekend and the last weekday, according to a validated recall method [29]. Multi-morbidity was defined as suffering from two or more morbidities [30]. Information on smoking status was based on self-report and participants were classified as never smokers, former smokers, or current smokers [31]. The body mass index (BMI) was defined as the body mass (weight) measured in kilograms divided by the square of the body height measured in meters. Frailty was defined according to the five criteria proposed by Fried et al. [32] in a slightly modified way, depending on the availability of information [33]. The glomerular filtration rate (eGFR) was estimated from serum creatinine as an indicator of renal function [34]. Use of medications and supplements ingested in the last seven days was collected through a database supported computer software (IDOM, Instrument for Databased Assessment Of Medication) [35], together with the mode, dosage and frequency of ingestion [36]. The micronutrient composition of supplements was available from a database established by staff of the Helmholtz Zentrum München. Polypharmacy was defined as the use of ≥5 medications, taken regularly and prescribed (without herbal or homeopathic medications).

### 2.7. Statistical Analysis

Descriptive statistics for categorical variables were expressed as frequencies and percentages. Due to non-normally distributed concentrations, serum levels of nutritional biomarkers were expressed as median and interquartile range (IQR), from the first (Q1) to the third quartile (Q3). When biomarker levels fell below the cut-offs for subclinical micronutrient deficiency, micronutrient status was categorized as ‘subclinical deficiency’ for the respective micronutrient. Prevalence estimates of subclinical micronutrient deficiency were reported with weighted percentages, using the Bavarian population per 31 December 2015 for age and sex standardization (www.statistikdaten.bayern.de/genesis/). Pearson’s chi-squared tests were conducted to assess sex and age differences in prevalence estimates.

To investigate predictors of subclinical micronutrient deficiencies, we defined subclinical micronutrient deficiency as the dependent variable and predictors as the categorical independent variables. The category with the ‘lowest risk’ was considered as reference for each independent variable (e.g., male sex, 65–74 years, never smoker etc.). Participants with missing information in one or more variables were excluded, except for frailty, which had a high number of missing values (*n* = 84). To avoid deletion of numerous information-rich participants, all participants with missing frailty status were considered as a separate category of the frailty variable. The analysis was performed separately for 25OHD, folate, vitamin B_12_ and iron, according to the following scheme. We started with a binary logistic regression, for each of the potential predictors, by calculating unadjusted odds ratios (ORs) and 95% confidence intervals (CIs). Variables that were significant at *p* < 0.25 were considered for inclusion in the multiple logistic regression model. Sex and age groups were forced into every model. As simultaneous intake of multiple supplements is frequent among older adults, we considered, for each nutritional biomarker, only supplement intake of the corresponding micronutrient. The final model, with variables significant at *p* < 0.05, resulted from a stepwise selection procedure, which is a combination of the forward and backward selection techniques. Model estimates are presented as fully adjusted ORs along with 95% CIs. All analyses were performed using the statistical software package, SAS version 9.4 (SAS Institute Inc., Cary, NC, USA).

## 3. Results

### 3.1. Baseline Characteristics of Study Participants

A description of baseline characteristics is shown in Table 1, stratified by sex. A total of *n* = 1079 individuals, aged 65 to 93 years, participated in the study, including 542 (50.2%) women. Overall, 47.7% of participants were physically inactive and 30.2% had a BMI ≥ 30 kg/m^2^, indicating obesity. Current smokers accounted for less than 5% of the population. Only 4.6% of all individuals were frail, but 37.9% were pre-frail. The prevalence of older adults having one or at least two diseases was 24.7% and 66.8%, respectively. Noticeable sex differences were observed for the variables, family status, educational level and alcohol consumption.

Seniors regularly using supplements containing vitamin D, folic acid, vitamin B_12_ and iron accounted for 6.7%, 9.5%, 8.9% and 2.8% of the male population, and 19.2%, 12.0%, 12.2% and 3.9% of the female population, respectively. In these regular users, the median amounts of vitamin D, folic acid, vitamin B_12_ and iron consumed from these supplements per day were, for men, 6.2 µg (IQR: 3.6–10.0), 400.0 µg (IQR: 200.0–714.3), 3.3 µg (IQR: 1.8–10.0) and 5.0 mg (IQR: 1.7–35.0), and for women 10.0 µg (IQR: 5.0–20.0), 300.0 µg (IQR: 200.0–400.0), 3.0 µg (IQR: 1.5–9.0) and 4.0 mg (IQR: 3.5–5.0), respectively.

Serum concentrations of 25OHD, folate, vitamin B_12_ and iron were measured in *n* = 1040, *n* = 1043, *n* = 1044 and *n* = 1050 KORA-Age individuals, respectively. Accordingly, a total of *n* = 39 (25OHD), *n* = 36 (folate), *n* = 35 (vitamin B_12_) and *n* = 29 individuals (iron) had missing values in serum nutritional biomarker measurements. Using Pearson’s chi-squared tests, KORA-Age individuals with missing micronutrient values were more likely to be very old (85–93 years); to live alone, to be divorced or widowed; to be less active or inactive; to be alcohol abstainers; to have a normal BMI (18.5 ≤ BMI < 25 kg/m^2^) and to have a low kidney function (eGFR < 60 mL/min/1.73 m^2^) (data not shown).

### 3.2. Prevalence of Subclinical Micronutrient Deficiency

Serum concentrations of nutritional biomarkers and total age- and sex-standardized prevalence of subclinical micronutrient deficiencies are presented in Table 2, stratified by sex.

Median concentrations of 25OHD, folate, vitamin B_12_ and iron were 48.3 nmol/L (IQR: 31.4–69.6), 24.5 nmol/L (IQR: 18.2–33.3), 277 pmol/L (IQR: 214–376) and 16.1 µmol/L (IQR: 13.1–20.0), respectively.

More than half of KORA-Age participants (52.0%) had low 25OHD concentrations. In season-specific analyses, the prevalence of a subclinical vitamin D deficiency was 60.9% in spring (February–May), 46.9% in summer (June–August) and 45.4% in fall (September–November). The prevalence of a subclinical vitamin B_12_ deficiency (27.3%) was also high. Approximately 10% of individuals had low levels of iron and folate. In sex-specific analyses, the prevalence of subclinical deficiencies were more common in women for 25OHD (59.0% vs. 44.4%) and folate (9.4% vs. 8.0%) and in men, for vitamin B_12_ (28.5% vs. 26.0%) and iron (13.5% vs. 8.4%). Using Pearson’s chi-squared tests, these sex differences in prevalence were significant for 25OHD and iron (*p* < 0.001).

**Figure 1:**
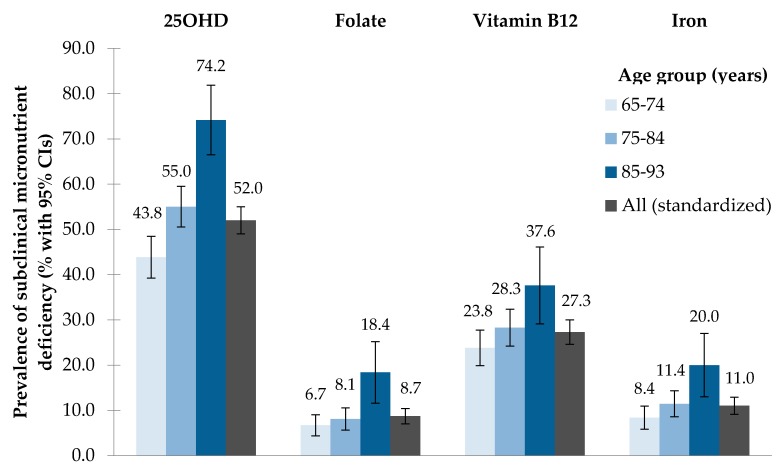
Prevalence of subclinical micronutrient deficiencies by age groups, based on serum biomarkers in KORA-Age 2008/2009 (25OHD = 25-hydroxyvitamin D; CI = confidence interval).

The prevalence of subclinical micronutrient deficiencies, by age groups, is shown in Figure 1. The prevalence increased gradually with age for all micronutrients. Specifically, the proportion of participants with serum 25OHD <50 nmol/L increased from 43.8% in the age group 65–74 years, to 74.2% in the age group 85–93 years. In Pearson’s chi-squared tests, divergences between age groups were significant for all biomarkers (25OHD: *p* < 0.001; folate: *p* < 0.001; vitamin B_12_: *p* = 0.008; iron: *p* = 0.001).

### 3.3. Predictors of Subclinical Micronutrient Deficiency

As illustrated in Supplementary Tables S2 and S3, 15 variables were tested in a binary logistic regression for the association between subclinical micronutrient deficiencies and each of the potential predictors, plus the season of blood collection for 25OHD. Besides sex and age groups, common potential predictors with *p*-values < 0.25 included educational level, nutritional status (Nutrition Score), physical activity, alcohol consumption, frailty and use of micronutrient-containing supplements.

The final results of the multiple logistic regression analyses are shown in Table 3. Common predictors that were significantly (*p* < 0.05) associated with low levels of several micronutrients were identified. Seniors aged 85 and over had two times higher odds of having low 25OHD (OR = 2.2, 95% CI 1.3–3.8, *p* = 0.003), low folate (OR = 2.3, 95% CI 1.2–4.3, *p* = 0.011) and low vitamin B_12_ levels (OR = 2.0, 95% CI 1.2–3.2, *p* = 0.004) compared to their 65–74-year old counterparts. Physical inactivity remained significantly associated with low 25OHD (OR = 1.6, 95% CI 1.2–2.2, *p* = 0.001), low folate (OR = 2.0, 95% CI 1.2–3.4, *p* = 0.006) and low vitamin B_12_ levels (OR = 1.4, 95% CI 1.0–1.8, *p* = 0.042). Both pre-frailty and frailty were strong independent correlates of subclinical 25OHD and iron deficiency. Frailty increased four-fold, the odds of a subclinical iron deficiency, compared to non-frail individuals (OR = 4.2, 95% CI 1.6–10.2, *p* = 0.002). Moreover, non/irregular users of supplements, containing specific micronutrients, had four to five times higher odds of having low 25OHD (OR = 4.8, 95% CI 3.1–7.6, *p* < 0.001), low folate (OR = 3.9, 95% CI 1.4–16.1, *p* = 0.024) and low vitamin B_12_ levels (OR = 4.7, 95% CI 2.5–10.2, *p* < 0.001), compared to regular users.

A few predictors remained independently associated with low levels of specific micronutrients only (Table 3). Springtime, female sex and obesity were significantly associated with a subclinical 25OHD deficiency. Former smokers had lower odds of having low 25OHD levels, compared to non-smokers. Polypharmacy was associated with better vitamin B_12_ levels. Regarding folate, drinkers of ≥20 g alcohol per day had lower odds of having low folate levels than non-drinkers. Finally, having a moderate/major and even low risk for malnutrition (as assessed by the GNRI), male sex and polypharmacy were associated with an increased risk for low iron levels.

## 4. Discussion

The determination of the magnitude and predictors of subclinical micronutrient deficiency in KORA-Age older adults revealed that the prevalence of subclinical vitamin D and B_12_ deficiencies were high. Very old age, physical inactivity, frailty and no/irregular use of micronutrient-containing supplements were common predictors of subclinical micronutrient deficiencies.

### 4.1. Prevalence of Subclinical Micronutrient Deficiency

Direct comparison of our findings with other studies is hampered by the lack of international consensus as to which nutritional biomarker (biochemical and/or functional), which assay methodology, and which cut off point should be used to define a subclinical micronutrient deficiency in older adults [23,37]. Nevertheless, our population-based data is in line with previous studies that have suggested that subclinical vitamin D and B_12_ deficiencies are prevalent public health problems among older adults [12,15,38,39,40,41,42].

In the present study, low 25OHD levels were found in more than half (52.0%) of KORA-Age participants, and were more frequent in women (59.0%) than in men (44.4%). These findings lie in the range of national estimates from selected European countries that have used 25OHD < 50 nmol/L as the cut-off, including Germany (women 69.9%, men 62.9%) [12], Austria (women 62.3%, men 64.8%) [38], France (women 42.2%, men 36.5%) [39] and England (women 57.0%, men 49.0%) [15]. 

Around one out of four participants (27.3%) had a low vitamin B_12_ concentration in the KORA-Age study population. In line with our data, a subclinical vitamin B_12_ deficiency has been reported in 10–15% of older adults aged 60 and over in the USA [40,41], and even in 23–35% of older adults aged 80 and over in the USA [42].

The prevalence of subclinical deficiencies for both serum folate and serum iron (~10%) was less of a concern in the studied older adults. Recent studies in the USA [43] and in Brazil [44] pointed to a prevalence of <5% for folate deficiency among older adults aged 60 and over, though it is important to note that these countries have adopted national folic acid fortification policies. For serum iron, comparable studies in older adults are lacking; other studies have focused on identifying more advanced cases of iron deficiency or anemia, or they have used, in combination with serum iron or without, other functional nutritional biomarkers of iron status, such as transferrin saturation, serum ferritin, or blood hemoglobin measurements [45,46,47]. In these studies, the prevalence of iron deficiency or anemia was only moderate (≤11%) in the older population.

Risk factors for subclinical micronutrient deficiency among older adults include: (i) decreased energy needs due to a loss of lean body mass and a decline in physical activity; (ii) age-associated physiological changes that affect absorption and utilization of micronutrients (e.g., decreased renal production of the active vitamin D form by the aging kidney [8]), and finally (iii) the presence of multiple chronic diseases, nutrient-drug interactions and variable diet quality, which may affect micronutrient requirements [9,10]. To identify older adults who would benefit most from subclinical micronutrient deficiency screening, we next discuss other factors that are associated with subclinical micronutrient deficiencies among older adults.

### 4.2. Predictors of Subclinical Micronutrient Deficiency

#### 4.2.1. Common Predictors of Subclinical Micronutrient Deficiency

##### Very Old Age

Seniors aged 85 and over were two times more likely to have low 25OHD, low folate and low vitamin B_12_ levels compared to their younger counterparts. It is well known that micronutrient inadequacies increase beyond 65 years [37,48], and even more in octogenarians and older [15,42].

The efficiency of producing vitamin D in the skin decreases with age [8], and may be coupled to age-related factors that limit sunlight exposure, such as being more housebound. A subclinical vitamin B_12_ deficiency in older adults is mainly caused by inadequate dietary intake and malabsorption. Chronic atrophic gastritis (a chronic inflammation causing loss of the gastric acid-producing cells, which is prevalent in ~30% of older adults) and the intake of drugs, such as proton pump inhibitors, histamine receptor 2 antagonists and biguanides, affect the secretion of gastric acid, thereby preventing the release of vitamin B_12_ from foods. Other contributing risk factors for low vitamin B_12_ levels in older adults include *Helicobacter pylori* infection and intestinal bacterial overgrowth [5,23]. An insufficient folate status in older adults seems to be mainly due to poor diet [49].

##### Physical Inactivity

Physical inactivity was associated with low 25OHD, folate and vitamin B_12_ concentrations. In epidemiological studies on vitamin D status, physical activity is often used as a proxy for time spent outdoors and indirectly for sunlight exposure. Accordingly, housebound seniors and those spending less time doing outdoor physical activity have decreased 25OHD levels [16]. Not surprisingly, being physically active was associated with higher 25OHD levels in our study. It has to be noted that our variable probably only gives a rough estimate of time spent outside, as it was not possible to distinguish outdoor from indoor activities.

Brock et al. found a positive association between both outdoor and indoor physical activities and 25OHD, suggesting that physical activity per se may also be a surrogate for better general health [50]. This may explain the findings for folate and vitamin B_12_. Prospective studies are needed to confirm a potential physiological link between physical activity and micronutrient concentrations.

##### Frailty

Pre-frail and frail individuals had a higher risk for subclinical 25OHD and iron deficiency. Prior cross-sectional and prospective studies among KORA-Age participants found low 25OHD levels to be associated with prevalent (pre-)frailty [33], incident pre-frailty and pre-frailty/frailty combined [51]. To our knowledge, the relationship between low serum iron levels and frailty occurrence has not been extensively investigated, yet Pires Corona et al. found that anemic older adults with low hemoglobin levels were more likely to be frail [52].

Although our findings suggest that (pre-)frailty may be a predictor of a subclinical micronutrient deficiency, it has also been suggested that poor nutrition itself can favor the development of frailty [53].

##### No/Irregular Use of Supplements

No/irregular use of supplements was a strong common predictor of subclinical 25OHD and folate and vitamin B_12_ deficiencies. Prior studies in older adults have found that supplement use is a major predictor of vitamin D status [14,17] and is linked to higher vitamin B_12_ and folate levels [54,55]. Although 44.7% of KORA-Age participants used supplements containing vitamins or minerals [36], only around 10% took supplements containing vitamin D, folate and vitamin B_12_, respectively. As shown previously [56,57], optimal serum micronutrient levels, such as vitamin D, folate and vitamin B_12_, can be maintained by supplementation. The possibility that regular supplementation with these specific micronutrients might help older adults in satisfying their requirements, and prevent chronic diseases via the correction of low levels, as found earlier [56], is of major interest and could stimulate research on biological pathways that link supplement intake, micronutrient status and disease state.

No/irregular use of iron-containing supplements was not significantly associated with a subclinical iron deficiency in our study. It is important to note that there are concerns about the adverse effects of iron-containing supplements [6], including nausea, epigastric discomfort and constipation, all of which are dose-related. In case of iron deficiency anemia, it is recommended to seek consultation from a physician, in order to use appropriately dosed iron-containing supplements.

#### 4.2.2. Further Predictors of Subclinical Micronutrient Deficiency

##### Vitamin D (Springtime, Female Sex, Obesity, Former Smoking)

In line with previous studies among older adults, we found that springtime, female sex and obesity were significantly associated with low 25OHD levels [15,16,17]. Seasonal variations in 25OHD status are well known. During cold and dark months, older adults may spend more daylight hours indoors, resulting in less sun exposure. Furthermore, in Germany, from October to March, UVB radiation is too low to induce vitamin D synthesis by the skin [58]. Sex differences may be due to unaccounted confounders, such as clothing style, use of sun protection, and to dietary intake. Concerning the inverse association with obesity, it has been suggested that vitamin D is accumulated in adipose tissue, resulting in decreased bioavailability and lower circulating 25OHD levels in the blood of obese persons [59].

The investigation of smoking status yielded inconsistent results. In studies classifying former smoking as a separate category [15,60], former smoking was not found to be a significant predictor of low 25OHD levels. In our data, former smoking was associated with higher 25OHD levels than never smokers. Schwab et al. showed that former smokers, among KORA-Age individuals, were two times more likely to ingest vitamin and mineral-containing supplements compared to never smokers [36]. It is therefore conceivable that smoking cessation went along with improved diet and lifestyle habits that increased vitamin D intake. These unaccounted factors, such as improved intake of foods rich in micronutrients or longer times spent outdoors, may have confounded the observed association. Furthermore, there is always the possibility for a type I error due to multiple comparisons.

##### Vitamin B_12_ (No Polypharmacy)

Some drugs reduce absorption or affect the metabolism of vitamin B_12_ via known mechanisms (such as proton pump inhibitors or histamine receptor 2 antagonists) or unknown mechanisms (such as metformin) [23]. Conversely, we found that polypharmacy was associated with sufficient vitamin B_12_ levels. Differentiating between the different types of medications ingested was outside the scope of this analysis, but it would be interesting to find out which medication(s) may positively impact on vitamin B_12_ status.

##### Folate (Alcohol Consumption)

Contra-intuitively, drinkers of ≥20 g alcohol per day had higher folate levels than abstainers. The consumption of ≥20 g of alcohol is equivalent to ≥0.5 L beer, ≥0.2 L wine or ≥3 small spirits of 2 cL [61], and was more frequent in men (*n* = 246) than women (*n* = 61) (Table 1). Differentiating between alcohol levels revealed that the majority of these female (*n* = 54) and male drinkers (*n* = 163) used between ≥20 and <40 g/day, which is still considered as moderate drinking for men. A lower proportion of men were high (between ≥40 to <60 g/day, *n* = 60) to heavy drinkers (>60 g/day, *n* = 23), respectively. Our variable category thus mostly covered moderate to high drinkers, and only a few heavy drinkers.

In observational epidemiological studies in humans, folate deficiency has been shown to be common in excessive alcohol drinkers and alcoholics [62], suggesting that alcohol consumption may affect folate status preponderantly at higher alcohol doses. Also, light to moderate alcohol consumption is known to be associated with a reduction in all-cause mortality [61]. It is thus possible that the dose at which alcohol was consumed in our study—mostly moderate amounts—was not harmful, but had a positive effect on health in general, thereby possibly affecting folate status. Possible mechanisms involved need further investigation. Nonetheless, caution is warranted as the observed association with alcohol use might be confounded by other factors that influence both traits. Another possible explanation for this counter-intuitive finding is the selection bias associated with the follow-up design of the KORA-Age study, whereby more health-interested individuals involved in former KORA studies, and probably fewer sick individuals with reduced alcohol consumption levels, participated.

##### Iron (Male Sex, GNRI, Polypharmacy)

Participants with a moderate/major and even low risk for malnutrition (GNRI) were at higher risk for low iron levels. The GNRI is a nutrition-related risk index that can predict the risk for morbidity and mortality in hospitalized older adults, in relation to malnutrition-associated pathologies. Our finding suggests that the GNRI may be a marker for subclinical iron deficiency in older adults. However, as the GNRI has primarily been established for use in hospitalized older adults, it may not necessarily be appropriate among community-dwelling older adults. Further research is warranted in this regard [26].

Men were at higher odds of having low iron levels, despite having higher median iron concentrations than women (Table 2). Sex divergence may be due to the use of a higher cut-off for men, as recommended in the literature [24]. Moreover, serum iron may increase after the ingestion of iron-containing foods [6], suggesting that sex differences may also be due to unequal dietary intakes. Finally, polypharmacy was inversely associated with iron status. Iron status in older adults is known to be affected by a number of medications [6], including antacids and proton pump inhibitors. Knowing which medication type may reduce iron levels is of interest from a prevention point of view and needs further research.

### 4.3. Strengths and Weaknesses

The KORA-Age study is singular in the sense that it includes a large (>1000 participants) and socio-demographically representative sample of old, and even very old, adults in Germany. Caution is warranted when generalizing findings on a national level, as possible regional differences in eating habits and latitude may exist. The assessment of multiple predictors of subclinical micronutrient deficiency was rendered possible by the extensive health assessment of participants and the quality of the KORA-Age data, which has been collected by trained interviewers and systematically controlled before entry into the KORA-Age database.

The use of biomarkers of micronutrient status is both a strength and a limitation of our study. Nutritional biomarkers may not necessarily reflect the amount of micronutrient intake from foods, and may be affected by multi-morbidity, inflammation and nutrient-drug interactions, especially in older adults. Nevertheless, biomarkers relate to the nutritional status of micronutrients after absorption in the body, and can directly reflect subclinical deficiency at the blood level.

Certain laboratory procedures, used for the assessment of micronutrient status were not the ‘gold standard’, e.g., the immunoassay procedure used in this study versus the commonly used *Lactobacillus rhamnosus* microbiological assay for measuring serum folate levels [22]. Also, the use of serum iron levels for the evaluation of iron status may be misleading because they are subject to diurnal rhythms and increase after the ingestion of iron-containing foods. However, serum iron concentrations indicate the adequacy of the iron supply to developing red blood cells [6], and may be used as a screening tool for a subclinical deficiency, as opposed to an established clinical deficiency. To identify more advanced cases of iron deficiency or anemia, additional measurements that use functional nutritional biomarkers are essential, including serum/plasma soluble transferrin receptor, serum/plasma ferritin, or blood hemoglobin measurements [22].

Another limitation arises from the cross-sectional design of this analysis, which precludes any direct cause-effect relationships between subclinical micronutrient deficiency and predictors. Also, causality cannot be definitively proven, owing to the possible correlation with other unknown or unmeasured predictors. Yet, a causal effect in one or the other direction is possible and has to be further examined in prospective studies.

## 5. Conclusions

Using serum nutritional biomarkers, determination of the magnitude of subclinical vitamin D, folate, vitamin B_12_ and iron deficiencies, among KORA-Age older adults, revealed that more than half of individuals had low 25OHD levels and more than a quarter had low vitamin B_12_ levels. The prevalence of subclinical deficiencies for both folate and iron (~10%) were less of a concern. Very old age, physical inactivity, frailty and no/irregular use of micronutrient-containing supplements were identified as common predictors of subclinical micronutrient deficiencies in the studied older adults.

Our findings provide further rationale for screening subgroups at high-risk for subclinical micronutrient deficiencies. The possibility that regular and appropriately dosed micronutrient supplementation might help older adults otherwise unable to follow dietary guidelines in satisfying their requirements, and prevent chronic diseases via the correction of low micronutrient levels, is of major interest and could stimulate research on biological pathways that link supplement intake, micronutrient status and disease state.

## Figures and Tables

**Table 1:** Baseline characteristics of older adults in KORA-Age 2008/2009, stratified by sex.

Baseline Characteristics	All (*n* = 1079)		Men (*n* = 537)		Women (*n* = 542)	
	*n*	%	*n*	%	*n*	%
**Season of blood collection**						
**Months of blood collection ^a^**						
February–May	440	42.3	242	46.2	198	38.4
June–August	360	34.6	173	33	187	36.2
September–November	240	23.1	109	20.8	131	25.4
**Socio-demographic factors**						
**Age groups (years)**						
65–74	457	42.4	233	43.4	224	41.3
75–84	486	45	243	45.3	243	44.8
85–93	136	12.6	61	11.4	75	13.8
**Family status ^b^**						
Living with a partner	663	62.1	424	79.3	239	44.9
Living alone, divorced or widowed	404	37.9	111	20.8	293	55.1
**Educational level (years)**						
Medium to high (10 to 17)	854	79.2	490	91.3	364	67.2
Low (8 to 9)	225	20.9	47	8.8	178	32.8
**Lifestyle factors**						
**Nutritional status**						
Geriatric Nutritional Risk Index (GNRI) ^c^						
No risk (>98)	960	92.3	482	92.3	478	92.3
Low risk (92 to 98)	59	5.7	29	5.6	30	5.8
Moderate or major risk (<92)	21	2	11	2.1	10	1.9
Nutrition Score (SCREEN II) ^d^						
Low risk (41 to 48)	408	38.3	239	44.8	169	31.7
Medium risk (36 to <41)	377	35.4	181	34	196	36.8
High risk (<36)	281	26.4	113	21.2	168	31.5
**Physical activity ^e^**						
Very active or moderately active	564	52.3	304	56.6	260	48.1
Less active or inactive	514	47.7	233	43.4	281	51.9
**Alcohol consumption (g/day) ^f^**						
0	393	36.6	126	23.5	267	49.7
>0 to <20	373	34.8	164	30.6	209	38.9
≥20	307	28.6	246	45.9	61	11.4
**Smoking status**						
Never smoker	617	57.2	204	38	413	76.2
Former smoker	413	38.3	303	56.4	110	20.3
Current smoker	49	4.5	30	5.6	19	3.5
**Health factors**						
**Body mass index (BMI) (kg/m^2^)**						
Normal (18.5 to <25)	228	21.1	99	18.4	129	23.8
Overweight (25 to <30)	525	48.7	281	52.3	244	45
Obese (≥30)	326	30.2	157	29.2	169	31.2
**Frailty ^g^**						
Non-frail	572	57.5	290	57.4	282	57.6
Pre-frail	377	37.9	194	38.4	183	37.4
Frail	46	4.6	21	4.2	25	5.1
**Polypharmacy (≥5 medications)**						
No	712	66	356	66.3	356	65.7
Yes	367	34	181	33.7	186	34.3
**eGFR (mL/min/1.73 m^2^)**						
Normal (≥60)	709	65.7	366	68.2	343	63.3
Low (<60)	370	34.3	171	31.8	199	36.7
**Multi-morbidity ^h^**						
No disease	91	8.5	56	10.5	35	6.6
One disease	263	24.7	138	25.9	125	23.4
Two or more diseases	712	66.8	338	63.5	374	70
**Use of supplements**						
Vitamin D						
Regular intake	140	13	36	6.7	104	19.2
No/irregular intake	939	87	501	93.3	438	80.8
Folic acid						
Regular intake	116	10.8	51	9.5	65	12
No/irregular intake	963	89.3	486	90.5	477	88
Vitamin B_12_						
Regular intake	114	10.6	48	8.9	66	12.2
No/irregular intake	965	89.4	489	91.1	476	87.8
Iron						
Regular intake	36	3.3	15	2.8	21	3.9
No/irregular intake	1043	96.7	522	97.2	521	96.1

SCREEN II: Seniors in the Community Risk Evaluation for Eating and Nutrition, version II; eGFR: estimated glomerular filtration rate; number of missing values: ^a^ 39, ^b^ 12, ^c^ 39, ^d^ 13, ^e^ 1, ^f^ 6, ^g^ 84, ^h^ 13.

**Table 2:** Biomarker concentrations and prevalence of subclinical micronutrient deficiencies by sex in KORA-Age 2008/2009.

Nutritional Biomarkers		Biomarker Concentrations (Percentile Scale)									Prevalence of Subclinical Deficiency		
	*n*	Min	1st	10th	Q1	Median	Q3	90th	99th	Max	%	% (Standardized *)	95% CI
**All**													
25OHD [nmol/L]	1040	3.7	7.7	21.3	31.4	48.3	69.6	93.1	136.3	174.7	52.5	52.0	49.0–55.0
Folate [nmol/L]	1043	5.8	9.0	14.0	18.2	24.5	33.3	45.3	45.3	90.6	8.7	8.7	7.0–10.4
Vitamin B_12_ [pmol/L]	1044	66	104	173	214	277	376	523	1476	1476	27.5	27.3	24.6–30.0
Iron [μmol/L]	1050	3.4	5.2	10.0	13.1	16.1	20.0	22.8	32.9	40.5	11.1	11.0	9.1–12.9
**Men**													
25OHD [nmol/L]	524	3.7	3.7	22.5	36.2	51.8	75.0	96.8	145.0	174.7	46.4	44.4	40.2–48.6
Folate [nmol/L]	524	5.8	10.2	14.4	17.9	23.8	31.3	45.3	45.3	63.9	8.2	8.0	5.7–10.3
Vitamin B_12_ [pmol/L]	525	68	104	170	210	263	355	494	1441	1476	29.3	28.5	24.6–32.4
Iron [μmol/L]	527	3.4	5.7	10.7	13.6	16.8	19.9	24.2	33.7	40.5	14.2	13.5	10.6–16.4
**Women**													
25OHD [nmol/L]	516	3.7	8.7	20.6	27.7	45.3	65.9	86.4	119.8	156.3	58.7	59.0	54.8–63.2
Folate [nmol/L]	519	7.3	8.9	13.8	18.4	25.2	35.4	45.3	45.3	90.6	9.2	9.4	6.9–12.0
Vitamin B_12_ [pmol/L]	519	66	108	175	218	292	392	542	1476	1476	25.6	26.0	22.3–29.3
Iron [μmol/L]	523	3.6	5.0	9.3	12.4	15.6	18.8	22.0	28.6	38.0	8.0	8.4	6.0–10.8

25OHD = 25-hydroxyvitamin D; Q1: first quartile or 25th percentile; Q3: third quartile or 75th percentile; CI: confidence interval; * Ten-year-age-group and sex-standardized prevalence using the Bavarian population per 31/12/2015; cut-offs for subclinical micronutrient deficiency: <50 nmol/L (25OHD); <13.6 nmol/L (folate); <221 pmol/L (vitamin B_12_); men: <11.6 µmol/L, women: <9.0 µmol/L (iron).

**Table 3:** Fully adjusted ORs with 95% CIs for subclinical micronutrient deficiencies by categories of identified predictors: Final results from multiple logistic regression analyses in KORA-Age 2008/2009.

Predictor	Predictor Categories	Low 25OHD (*n* = 525)			Low Folate (*n* = 86)			Low Vitamin B_12_ (*n* = 283)			Low Iron (*n* = 106)		
		OR	95% CI	*p*	OR	95% CI	*p*	OR	95% CI	*p*	OR	95% CI	*p*
**Season of blood collection**													
Months of blood collection	February–May vs. June–August	2.1	1.5–2.8	<0.001	.	.	.	.	.	.	.	.	.
Months of blood collection	September–November vs. June–August	0.8	0.5–1.1	0.135	.	.	.	.	.	.	.	.	.
**Socio-demographic factors**													
Sex	Women vs. men	1.9	1.4–2.5	<0.001	0.8	0.5–1.4	0.459	0.8	0.6–1.1	0.231	0.4	0.3–0.7	<0.001
Age groups (years)	75–84 vs. 65–74	1.3	1.0–1.8	0.058	0.9	0.6–1.6	0.818	1.3	0.9–1.8	0.110	1.0	0.6–1.7	0.911
Age groups (years)	85–93 vs. 65–74	2.2	1.3–3.8	0.003	2.3	1.2–4.3	0.011	2.0	1.2–3.2	0.004	1.2	0.6–2.3	0.564
**Lifestyle factors**													
Nutritional status	GNRI: Low (92 to 98) vs. no risk (>98)	-	-	-	-	-	-	-	-	-	2.7	1.3–5.4	0.005
Nutritional status	GNRI: Moderate/major (<92) vs. no risk (>98)	-	-	-	-	-	-	-	-	-	4.0	1.2–12.0	0.015
Physical activity	Less active or inactive vs. very active or moderately active	1.6	1.2–2.2	0.001	2.0	1.2–3.4	0.006	1.4	1.0–1.8	0.042	-	-	-
Alcohol consumption (g/day)	>0 to <20 vs. 0	-	-	-	1.0	0.6–1.6	0.876	-	-	-	-	-	-
Alcohol consumption (g/day)	≥20 vs. 0	-	-	-	0.4	0.2–0.8	0.017	-	-	-	-	-	-
Smoking status	Current smoker vs. never smoker	0.8	0.4–1.5	0.516	-	-	-	-	-	-	-	-	-
Smoking status	Former smoker vs. never smoker	0.6	0.4–0.8	0.002	-	-	-	-	-	-	-	-	-
**Health factors**													
BMI (kg/m^2^)	Overweight (25 to <30) vs. normal (18.5 to <25)	0.9	0.7–1.4	0.763	-	-	-	-	-	-	-	-	-
BMI (kg/m^2^)	Obese (≥30) vs. normal (18.5 to <25)	1.8	1.2–2.6	0.005	-	-	-	-	-	-	-	-	-
Frailty	Missing value vs. non-frail	1.1	0.6–2.2	0.736	-	-	-	-	-	-	3.4	1.3–8.0	0.008
Frailty	Pre-frail vs. non-frail	1.8	1.3–2.5	<0.001	-	-	-	-	-	-	2.7	1.7–4.5	<0.001
Frailty	Frail vs. non-frail	2.5	1.2–5.4	0.022	-	-	-	-	-	-	4.2	1.6–10.2	0.002
Polypharmacy	Yes vs. no	-	-	-	-	-	-	0.5	0.4–0.7	<0.001	1.6	1.0–2.4	0.045
Use of supplements	Vitamin D: No/irregular intake vs. regular intake	4.8	3.1–7.6	<0.001	.	.	.	.	.	.	.	.	.
Use of supplements	Folic acid: No/irregular intake vs. regular intake	.	.	.	3.9	1.4–16.1	0.024	.	.	.	.	.	.
Use of supplements	Vitamin B_12_: No/irregular intake vs. regular intake	.	.	.	.	.	.	4.7	2.5–10.2	<0.001	.	.	.

25OHD = 25-hydroxyvitamin D; GNRI: Geriatric Nutritional Risk Index; BMI: body mass index; OR: odds ratio; CI: confidence interval; *p*: p-value; -: variable not significant at *p* < 0.05; .: not investigated (see Methods); cut-offs for subclinical micronutrient deficiency: <50 nmol/L (25OHD); <13.6 nmol/L (folate); <221 pmol/L (vitamin B_12_); men: <11.6 µmol/L, women: <9.0 µmol/L (iron).

## Supplementary Materials

The following are available online at www.mdpi.com/2072-6643/9/12/1276/s1, Figure S1: Flowchart of the KORA-Age 2008/2009 recruitment and retention profile, Table S1: Description of data collection and categorization methods for the investigated predictors in KORA-Age 2008/2009, Table S2: Unadjusted ORs with 95% CIs for subclinical micronutrient deficiencies by categories of potential predictors: Results from binary logistic regression analyses in KORA-Age 2008/2009, Table S3: Predictors with *p* < 0.25 in binary logistic regression which were entered into each multiple logistic regression model.

## Abbreviations

25OHD	25-hydroxyvitamin D
BMI	Body Mass Index
CI	Confidence interval
CVD	Cardiovascular disease
DEGS1	German Health Interview and Examination Survey for Adults
DGE	German Nutrition Society
eGFR	Estimated Glomerular Filtration Rate
GNRI	Geriatric Nutritional Risk Index
IDOM	Instrument for Databased Assessment Of Medication
IQR	Interquartile range
KORA	Cooperative Health Research in the Region of Augsburg
MONICA	Monitoring of Trends and Determinants in Cardiovascular Disease
NNR	Nordic Nutrition Recommendations
NVS II	Second German National Nutrition Survey
OR	Odds ratio
Q1	First quartile or 25th percentile
Q3	Third quartile or 75th percentile
SCREEN II	Seniors in the Community Risk Evaluation for Eating and Nutrition, version II
WHO	World Health Organization
