# Supplementary material for: Prevalence and Predictors of Subclinical Micronutrient Deficiency in German Older Adults: Results from the Population-Based KORA-Age Study
Source: Nutrients. 2017 Nov 23;9(12):1276. doi: 10.3390/nu9121276 (PMC5748727; doi:10.3390/nu9121276)
Supplement: Supplementary file 1 [file nutrients-09-01276-s001.pdf]

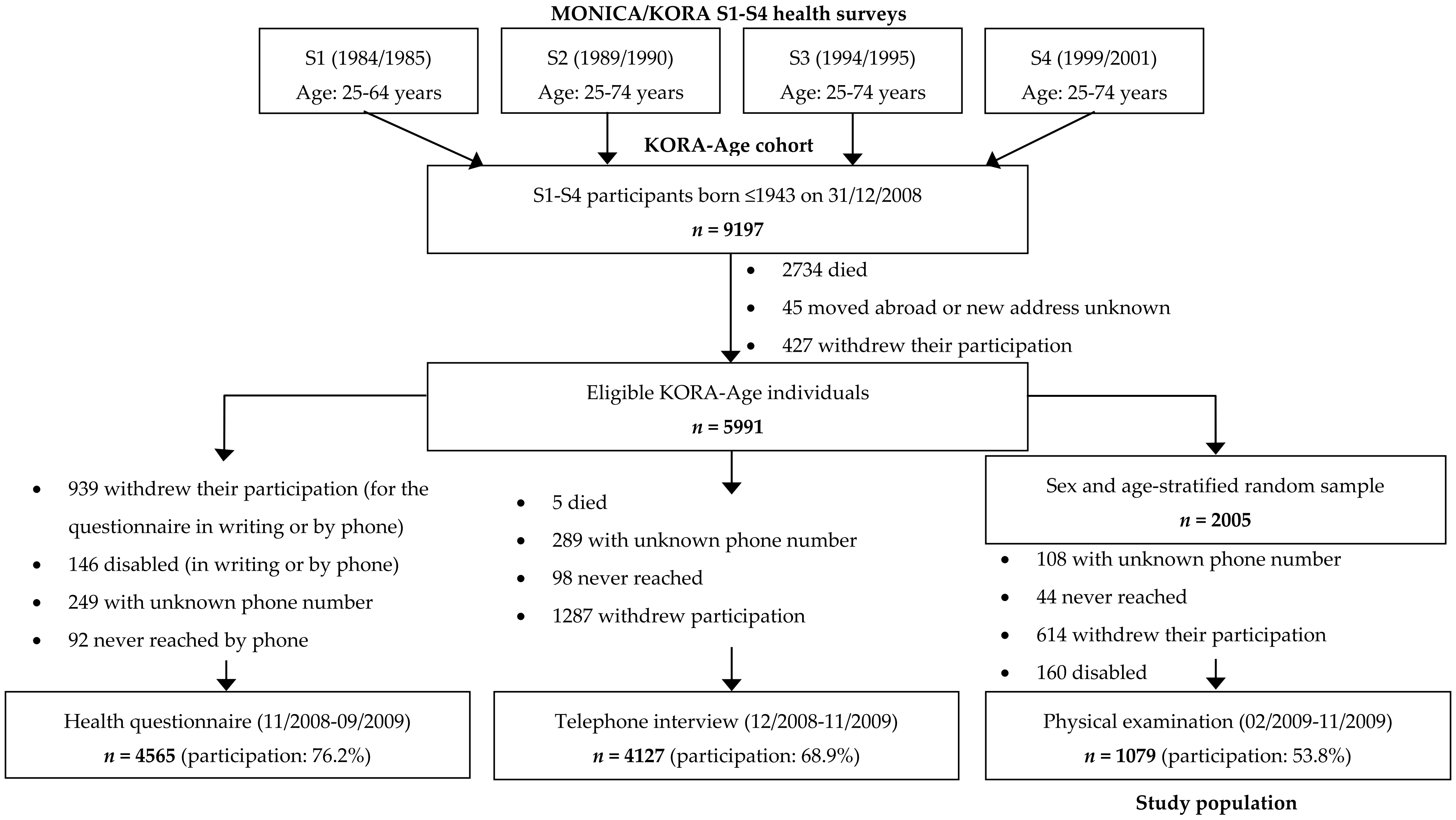


**Figure S1**: Flowchart of the KORA-Age 2008/2009 recruitment and retention profile.

**Table S1**: Description of data collection and categorization methods for the investigated predictors in KORA-Age 2008/2009.

| Predictor | Collection Method | Assessment Method | Categories |
|---|---|---|---|
| **Socio-demographic factors** | | | |
| Sex | Telephone interview | Short form of the Demographic Standards of the German Society of Epidemiology [1] | Men; women |
| Age groups (years) | Telephone interview | Same as sex | 65-74; 75-84; 85-93 |
| Family status | Telephone interview | Same as sex | Living with a partner; living alone, divorced or widowed |
| Educational level (years) | Telephone interview | Estimated by recording years of school completed | Medium to high (10 to 17); low (8 to 9) |
| **Lifestyle factors** | | | |
| Nutritional status (GNRI) | Physical examination | Derived by combining information on albumin level, weight and height, defined in [2] | No risk (>98); low risk (92 to 98); moderate/ major risk (<92) |
| Nutritional status (Nutrition Score) | Telephone interview | Short version of SCREEN II questionnaire covering eating habits (e.g. skipping meals, appetite, daily intake of fruits and vegetables) [3] | Low risk (41 to 48); medium risk (36 to <41); high risk (<36) |
| Physical activity | Telephone interview | Two four-category questions on time spent per week on sport activities in summer and winter, respectively [4] | Very active (regular sports in summer and winter for >2 hours/week in both seasons) or moderately active (regular sports for ~1 hour/week in at least one season); less active (irregular sports for ~1 hour/week in at least one season) or inactive (no sports in summer or winter) |
| Alcohol consumption (g/day) | Physical examination | Self-reported amount of alcoholic drinks consumed (beer, wine and spirits) based on the last weekend and the last weekday using a validated recall method [5] | 0; >0 to <20; ≥20 |
| Smoking status | Health questionnaire | Questions: 1) Have you smoked more than 100 cigarettes in your life? 2) Do you smoke cigarettes at the moment? 3) Do you smoke regularly or occasionally? [6] | Never smoker (if answer 'no' to question 1); ex-smoker (if answer 'yes' to question 1 but answer 'no' to question 2); current smoker (if answer 'regularly' or 'occasionally' to question 3) |

**Table S1**: *Cont.*

| Predictor | Collection Method | Assessment Method | Categories |
|---|---|---|---|
| **Health factors** | | | |
| BMI (kg/m$^2$) | Physical examination | Body weight in kilograms divided by squared height in meters | According to WHO: normal (18.5 to <25); overweight (25 to <30); obese (≥30). One individual with a BMI of 18.3 was added to the category normal (18.5 to <25). |
| Frailty | Physical examination | Adapted version of the frailty phenotype proposed by Fried et al. [7], defined by the presence of ≥3 criteria: weight loss (>5 kg in the past 6 months), exhaustion, low physical activity (by self-report), low walking speed and weakness (as measured by grip strength) | Non-frail (no criteria); pre-frail (1-2 criteria); frail (≥3 criteria) |
| Polypharmacy | Physical examination | Use of ≥5 medications, taken regularly and prescribed (without herbal or homeopathic medications) | No; yes |
| eGFR (mL/min/1.73m$^2$) | Physical examination | Estimated from serum creatinine (mg/dL) according to formulas described in [8] | Normal (≥60); low (<60) |
| Multi-morbidity | Health questionnaire + telephone interview | Suffering from ≥2 morbidities including hypertension, eye disease, heart disease, diabetes mellitus, joint disease, lung disease, gastrointestinal disease, mental disease, stroke, cancer, kidney disease, neurological disease, liver disease [9] | No disease; one disease; two or more diseases |
| Use of supplements | Physical examination | Use of medications and supplements ingested in the last 7 days collected through a database supported computer software (IDOM) [10], together with mode, dosage and frequency of ingestion [11]. Micronutrient composition of supplements available from a database established by Helmholtz Zentrum München staff [11]. | Regular intake; no/irregular intake |

GNRI: Geriatric Nutritional Risk Index; SCREEN II: Seniors in the Community Risk Evaluation for Eating and Nutrition, version II; BMI: body mass index; WHO: World Health Organization; eGFR: estimated glomerular filtration rate; IDOM: Instrument for Databased Assessment Of Medication

**Table S2**: Unadjusted ORs with 95% CIs for subclinical micronutrient deficiencies by categories of potential predictors: Results from binary logistic regression analyses in KORA-Age 2008/2009.

| Predictor | Predictor Categories | Low 25OHD | | | Low folate | | | Low vitamin $B_{12}$ | | | Low iron | | |
|---|---|---|---|---|---|---|---|---|---|---|---|---|---|
| | | OR | 95% CI | *p* | OR | 95% CI | *p* | OR | 95% CI | *p* | OR | 95% CI | *p* |
| **Season of blood collection** | | | | | | | | | | | | | |
| Months of blood collection | February-May vs June-August | 1.8 | 1.3 – 2.3 | <0.001 | . | . | . | . | . | . | . | . | . |
| Months of blood collection | September-November vs June-August | 0.9 | 0.7 – 1.3 | 0.713 | . | . | . | . | . | . | . | . | . |
| **Socio-demographic factors** | | | | | | | | | | | | | |
| Sex | Women vs men | 1.6 | 1.3 – 2.1 | <0.001 | 1.2 | 0.8 – 1.8 | 0.479 | 0.8 | 0.6 – 1.1 | 0.180 | 0.5 | 0.3 – 0.7 | 0.001 |
| Age groups (years) | 75-84 vs 65-74 | 1.6 | 1.2 – 2.0 | 0.001 | 1.2 | 0.7 – 2.0 | 0.494 | 1.3 | 0.9 – 1.7 | 0.124 | 1.5 | 1.0 – 2.3 | 0.075 |
| Age groups (years) | 85-93 vs 65-74 | 3.7 | 2.4 – 5.8 | <0.001 | 3.1 | 1.7 – 5.6 | <0.001 | 1.9 | 1.3 – 2.9 | 0.002 | 2.8 | 1.6 – 4.8 | <0.001 |
| Family status | Living alone, divorced or widowed vs living with a partner | 1.4 | 1.1 – 1.9 | 0.006 | 1.4 | 0.9 – 2.2 | 0.105 | 0.9 | 0.7 – 1.2 | 0.545 | 1.2 | 0.8 – 1.8 | 0.348 |
| Educational level (years) | Low (8 to 9) vs medium to high (10 to 17) | 1.7 | 1.2 – 2.3 | 0.001 | 1.4 | 0.8 – 2.2 | 0.217 | 1.3 | 0.9 – 1.7 | 0.161 | 1.0 | 0.6 – 1.7 | 0.850 |
| **Lifestyle factors** | | | | | | | | | | | | | |
| Nutritional status | GNRI: Low (92 to 98) vs no risk (>98) | 1.0 | 0.6 – 1.7 | 0.923 | 1.3 | 0.5 – 2.8 | 0.614 | 1.0 | 0.5 – 1.7 | 0.926 | 2.7 | 1.4 – 5.1 | 0.003 |
| Nutritional status | GNRI : Moderate/major (<92) vs no risk (>98) | 1.5 | 0.6 – 3.8 | 0.367 | 1.2 | 0.2 – 4.1 | 0.840 | 0.6 | 0.2 – 1.7 | 0.386 | 4.8 | 1.8 – 11.9 | 0.001 |
| Nutritional status | Nutrition Score: High (<36) vs low risk (41 to 48) | 1.5 | 1.1 – 2.0 | 0.019 | 2.0 | 1.2 – 3.5 | 0.012 | 1.2 | 0.8 – 1.7 | 0.393 | 1.7 | 1.1 – 2.8 | 0.016 |
| Nutritional status | Nutrition Score: Medium (36 to <41) vs low risk (41 to 48) | 1.2 | 0.9 – 1.7 | 0.135 | 1.2 | 0.7 – 2.1 | 0.484 | 1.3 | 1.0 – 1.8 | 0.076 | 0.7 | 0.4 – 1.1 | 0.112 |
| Physical activity | Less active or inactive vs very active or moderately active | 2.1 | 1.6 – 2.7 | <0.001 | 2.3 | 1.5 – 3.7 | <0.001 | 1.5 | 1.1-1.9 | 0.006 | 1.8 | 1.2 – 2.7 | 0.003 |
| Alcohol consumption (g/day) | >0 to <20 vs 0 | 0.8 | 0.6 – 1.0 | 0.056 | 0.9 | 0.5 – 1.4 | 0.571 | 1.1 | 0.8 – 1.5 | 0.612 | 0.9 | 0.6 – 1.5 | 0.763 |
| Alcohol consumption (g/day) | ≥20 vs 0 | 0.5 | 0.4 – 0.7 | <0.001 | 0.4 | 0.2 – 0.8 | 0.009 | 1.3 | 1.0 – 1.9 | 0.083 | 0.9 | 0.6 – 1.5 | 0.750 |
| Smoking status | Current smoker vs never smoker | 0.8 | 0.4 – 1.4 | 0.345 | 0.9 | 0.3 – 2.4 | 0.893 | 1.6 | 0.9 – 3.0 | 0.107 | 2.2 | 1.0 – 4.6 | 0.043 |
| Smoking status | Former smoker vs never smoker | 0.6 | 0.4 – 0.7 | <0.001 | 1.0 | 0.6 – 1.5 | 0.920 | 0.9 | 0.7 – 1.2 | 0.425 | 1.4 | 0.9 – 2.1 | 0.101 |

**Table S2**: *Cont.*

| Predictor | Predictor categories | Low 25OHD | | | Low folate | | | Low vitamin $B_{12}$ | | | Low iron | | |
|---|---|---|---|---|---|---|---|---|---|---|---|---|---|
| | | OR | 95% CI | *p* | OR | 95% CI | *p* | OR | 95% CI | *p* | OR | 95% CI | *p* |
| **Health factors** | | | | | | | | | | | | | |
| BMI (kg/m$^2$) | Overweight (25 to <30) vs normal (18.5 to <25) | 1.0 | 0.7 – 1.4 | 0.953 | 1.1 | 0.6 – 2.2 | 0.675 | 1.2 | 0.9 – 1.8 | 0.287 | 0.7 | 0.5 – 1.2 | 0.188 |
| BMI (kg/m$^2$) | Obese (≥30) vs normal (18.5 to <25) | 1.7 | 1.2 – 2.5 | 0.003 | 1.6 | 0.8 – 3.0 | 0.162 | 1.2 | 0.8 – 1.8 | 0.378 | 0.5 | 0.3 – 0.9 | 0.020 |
| Frailty | Missing value vs non-frail | 2.0 | 1.2 – 3.6 | 0.016 | 3.1 | 1.4 – 6.5 | 0.003 | 1.4 | 0.7 – 2.4 | 0.297 | 4.1 | 1.9 – 8.4 | <0.001 |
| Frailty | Pre-frail vs non-frail | 2.2 | 1.7 – 2.8 | <0.001 | 1.5 | 0.9 – 2.5 | 0.091 | 1.2 | 0.9 – 1.7 | 0.156 | 3.3 | 2.1 – 5.2 | <0.001 |
| Frailty | Frail vs non-frail | 2.9 | 1.6 – 5.8 | 0.001 | 3.6 | 1.5 – 7.7 | 0.002 | 1.6 | 0.8 – 2.9 | 0.158 | 6.9 | 3.2 – 14.2 | <0.001 |
| Polypharmacy | Yes vs no | 1.3 | 1.0 – 1.6 | 0.079 | 1.0 | 0.7 – 1.6 | 0.852 | 0.6 | 0.4 – 0.8 | 0.001 | 2.2 | 1.5 – 3.2 | <0.001 |
| eGFR (mL/min/1.73m$^2$) | Low (<60) vs normal (≥60) | 1.6 | 1.2 – 2.1 | 0.001 | 1.5 | 1.0 – 2.3 | 0.066 | 0.1 | 0.0 – 1.0 | 0.990 | 2.0 | 1.3 – 2.9 | 0.001 |
| Multi-morbidity | One disease vs no disease | 1.6 | 1.0 – 2.7 | 0.049 | 0.8 | 0.3 – 2.1 | 0.571 | 1.3 | 0.8 – 2.2 | 0.361 | 1.3 | 0.5 – 4.2 | 0.568 |
| Multi-morbidity | Two or more disease vs no disease | 2.4 | 1.5 – 3.8 | <0.001 | 1.3 | 0.6 – 3.1 | 0.571 | 1.0 | 0.6 – 1.8 | 0.861 | 2.5 | 1.1 – 7.3 | 0.051 |
| Use of supplements | Vitamin D: No/irregular intake vs regular intake | 3.4 | 2.3 – 5.2 | <0.001 | . | . | . | . | . | . | . | . | . |
| Use of supplements | Folic acid: No/irregular intake vs regular intake | . | . | . | 3.8 | 1.4 – 15.6 | 0.026 | . | . | . | . | . | . |
| Use of supplements | Vitamin $B_{12}$: No/irregular intake vs regular intake | . | . | . | . | . | . | 4.8 | 2.5 – 10.4 | <0.001 | . | . | . |
| Use of supplements | Iron: No/irregular intake vs regular intake | . | . | . | . | . | . | . | . | . | 4.1 | 0.9 – 73.7 | 0.164 |

25OHD=25-hydroxyvitamin D; GNRI: Geriatric Nutritional Risk Index; SCREEN II: Seniors in the Community Risk Evaluation for Eating and Nutrition, version II; BMI: body mass index; eGFR: estimated glomerular filtration rate; OR: odds ratio; CI: confidence interval; *p*: p-value; . : not investigated (see Methods); range of *n* for 25OHD: 1030-1040, folate: 1033-1043, vitamin $B_{12}$: 1034-1044, iron: 1040-1050; variables with $p < 0.25$ were selected for multiple logistic regression analysis; cut-offs for subclinical micronutrient deficiency: <50 nmol/L (25OHD); <13.6 nmol/L (folate); <221 pmol/L (vitamin $B_{12}$); men: <11.6 µmol/L, women: <9.0 µmol/L (iron)

**Table S3**: Predictors with $p < 0.25$ in binary logistic regression which were entered into each multiple logistic regression model

| Low 25OHD | Low folate | Low vitamin $B_{12}$ | Low iron |
|---|---|---|---|
| Months of blood collection | . | . | . |
| Sex* | Sex** | Sex* | Sex* |
| Age groups* | Age groups* | Age groups* | Age groups* |
| Family status | Family status | - | - |
| Educational level | Educational level | Educational level | - |
| - | - | - | Nutritional status (GNRI) |
| Nutritional status (Nutrition Score) | Nutritional status (Nutrition Score) | Nutritional status (Nutrition Score) | Nutritional status (Nutrition Score) |
| Physical activity | Physical activity | Physical activity | Physical activity |
| Alcohol consumption | Alcohol consumption | Alcohol consumption | - |
| Smoking status | - | Smoking status | Smoking status |
| BMI | BMI | - | BMI |
| Frailty | Frailty | Frailty | Frailty |
| Polypharmacy | - | Polypharmacy | Polypharmacy |
| eGFR | eGFR | - | eGFR |
| Multi-morbidity | - | - | Multi-morbidity |
| Use of supplements (Vitamin D) | . | . | . |
| . | Use of supplements (Folic acid) | . | . |
| . | . | Use of supplements (Vitamin $B_{12}$) | . |
| . | . | . | Use of supplements (Iron) |

25OHD=25-hydroxyvitamin D ; GNRI: Geriatric Nutritional Risk Index; BMI: body mass index; eGFR: estimated glomerular filtration rate; *: variables were forced in every model (**even if non-significant at $p < 0.25$); -: variable not significant at $p < 0.25$; . : not investigated (see Methods); cut-offs for subclinical micronutrient deficiency: <50 nmol/L (25OHD); <13.6 nmol/L (folate); <221 pmol/L (vitamin $B_{12}$); men: <11.6 µmol/L, women: <9.0 µmol/L (iron

## References


1. Ahrens, W.; Bellach, B.-M.; Jöckel, K.-H. [Measurement and quantification of sociodemographic characteristics in epidemiological studies]. German Society for Medical Informatics, Biometry and Epidemiology (GMDS). **1998**. Available online: https://dgepi.de/fileadmin/pdf/leitlinien/11_MessungUndQuantifizierungSoziodemographischerMerkmale_pdf2.pdf (accessed on 14 November 2017).
2. Bouillanne, O.; Morineau, G.; Dupont, C.; Coulombel, I.; Vincent, J.P.; Nicolis, I.; Benazeth, S.; Cynober, L.; Aussel, C. Geriatric Nutritional Risk Index: a new index for evaluating at-risk elderly medical patients. *Am J Clin Nutr* **2005**, *82*, 777-783.
3. Keller, H.H.; Goy, R.; Kane, S.L. Validity and reliability of SCREEN II (Seniors in the community: risk evaluation for eating and nutrition, Version II). *Eur J Clin Nutr* **2005**, *59*, 1149-1157. 10.1038/sj.ejcn.1602225.
4. Meisinger, C.; Lowel, H.; Thorand, B.; Doring, A. Leisure time physical activity and the risk of type 2 diabetes in men and women from the general population. The MONICA/KORA Augsburg Cohort Study. *Diabetologia* **2005**, *48*, 27-34. 10.1007/s00125-004-1604-3.
5. Doring, A.; Filipiak, B.; Stieber, J.; Keil, U. Trends in alcohol intake in a southern German population from 1984-1985 to 1989-1990: results of the MONICA Project Augsburg. *J Stud Alcohol* **1993**, *54*, 745-749.
6. Strobl, R.; Muller, M.; Emeny, R.; Peters, A.; Grill, E. Distribution and determinants of functioning and disability in aged adults--results from the German KORA-Age study. *BMC Public Health* **2013**, *13*, 137. 10.1186/1471-2458-13-137.
7. Fried, L.P.; Tangen, C.M.; Walston, J.; Newman, A.B.; Hirsch, C.; Gottdiener, J.; Seeman, T.; Tracy, R.; Kop, W.J.; Burke, G., *et al.* Frailty in older adults: evidence for a phenotype. *J Gerontol A Biol Sci Med Sci* **2001**, *56*, M146-156.
8. Inker, L.A.; Schmid, C.H.; Tighiouart, H.; Eckfeldt, J.H.; Feldman, H.I.; Greene, T.; Kusek, J.W.; Manzi, J.; Van Lente, F.; Zhang, Y.L., *et al.* Estimating glomerular filtration rate from serum creatinine and cystatin C. *N Engl J Med* **2012**, *367*, 20-29. 10.1056/NEJMoa1114248.
9. Kirchberger, I.; Meisinger, C.; Heier, M.; Zimmermann, A.K.; Thorand, B.; Autenrieth, C.S.; Peters, A.; Ladwig, K.H.; Doring, A. Patterns of multimorbidity in the aged population. Results from the KORA-Age study. *PLoS One* **2012**, *7*, e30556. 10.1371/journal.pone.0030556.
10. Mühlberger, N.; Behrend, C.; Stark, R.; Holle, R. [Database-supported identification and entry of drug data in health studies - experience with the IDOM software]. *Informatik Biometrie und Epidemiologie in Medizin und Biologie* **2003**, *34*, 601-611.
11. Schwab, S.; Heier, M.; Schneider, A.; Fischer, B.; Huth, C.; Peters, A.; Thorand, B. The use of dietary supplements among older persons in southern Germany - results from the KORA-age study. *J Nutr Health Aging* **2014**, *18*, 510-519. 10.1007/s12603-013-0418-8.


© 2017 by the authors. Submitted for possible open access publication under the terms and conditions of the Creative Commons Attribution (CC BY) license (http://creativecommons.org/licenses/by/4.0/).
